# Effect of EVAR on International Ruptured AAA Mortality—Sex and Geographic Disparities

**DOI:** 10.3390/jcm13092464

**Published:** 2024-04-23

**Authors:** C. Y. Maximilian Png, A. Alaska Pendleton, Martin Altreuther, Jacob W. Budtz-Lilly, Kim Gunnarsson, Chung-Dann Kan, Manar Khashram, Matti T. Laine, Kevin Mani, Christian C. Pederson, Sunita D. Srivastava, Matthew J. Eagleton

**Affiliations:** 1 Division of Vascular and Endovascular Surgery, Department of Surgery, Massachusetts General Hospital, Boston, MA 02114, USA; 2 Department of Vascular Surgery, St. Olavs Hospital, 7030 Trondheim, Norway; 3 Cardio-Thoracic and Vascular Surgery, Aarhus University Hospital, Skejby, 8200 Aarhus, Denmark; 4 Department of Surgical Sciences, Uppsala University, 75237 Uppsala, Sweden; 5 Department of Surgery, National Cheng Kung University Hospital, College of Medicine, National Cheng Kung University, Tainan City 701, Taiwan; kcd5086@gmail.com; 6 Department of Surgery, University of Auckland, Auckland 1010, New Zealand; 7 Department of Vascular Surgery, University of Helsinki and Helsinki University Hospital, 00029 Helsinki, Finland; matti.laine@hus.fi; 8 Department of Vascular Surgery, Aalborg University Hospital, 9000 Aalborg, Denmark

**Keywords:** EVAR, ruptured AAA, sex disparities, geographical disparities, vascular surgery

## Abstract

**Background**: We sought to investigate the differential impact of EVAR (endovascular aneurysm repair) vis-à-vis OSR (open surgical repair) on ruptured AAA (abdominal aortic aneurysm) mortality by sex and geographically. **Methods**: We performed a retrospective study of administrative data on EVAR from state statistical agencies, vascular registries, and academic publications, as well as ruptured AAA mortality rates from the World Health Organization for 14 14 states across Australasia, East Asia, Europe, and North America. **Results**: Between 2011–2016, the proportion of treatment of ruptured AAAs by EVAR increased from 26.1 to 43.8 percent among females, and from 25.7 to 41.2 percent among males, and age-adjusted ruptured AAA mortality rates fell from 12.62 to 9.50 per million among females, and from 34.14 to 26.54 per million among males. The association of EVAR with reduced mortality was more than three times larger (2.2 vis-à-vis 0.6 percent of prevalence per 10 percentage point increase in EVAR) among females than males. The association of EVAR with reduced mortality was substantially larger (1.7 vis-à-vis 1.1 percent of prevalence per 10 percentage point increase in EVAR) among East Asian states than European+ states. **Conclusions**: The increasing adoption of EVAR coincided with a decrease in ruptured AAA mortality. The relationship between EVAR and mortality was more pronounced among females than males, and in East Asian than European+ states. Sex and ethnic heterogeneity should be further investigated.

## 1. Introduction

Studies of abdominal aortic aneurysms (AAAs) have shown decreasing trends in mortality rates in both sexes, across populations in Australia, Europe, and North America in the last two decades [1,2,3]. Meanwhile, endovascular aneurysm repair (EVAR) has supplanted open surgical repair (OSR) as the most common modality of repair [4,5,6]. The increased use of EVAR has been suggested as one explanation for the reduction in AAA mortality [1].

The vast majority of current literature on AAA repair revolves around perioperative mortality, and although it is widely accepted that EVAR has significantly decreased rates of perioperative mortality (both for ruptured and non-ruptured aneurysms), the reduction in mortality and the longer term benefits have been questioned [7]. Moreover, the advent of EVAR has increased the pool of potential candidates for surgical repair. However, the impact of this on overall AAA mortality has yet to be studied.

Furthermore, it is worth noting that the majority of the AAA literature has been limited to predominantly Caucasian and male populations [1,2,3,5]. In line with EVARs being designed on the prototypical aorta of a white male, both non-Caucasian and female patients are far less likely to satisfy the EVAR instructions for use (IFU), with an odds ratio as low as 0.4 in females [8]. Furthermore, compared to Caucasians, Asians are almost three times more likely to have “hostile” aortic necks and have a higher likelihood of access-related complications due to smaller iliac artery diameters [9,10,11].

We thus sought to examine the relationship between EVAR and ruptured AAA mortality, considering differences by sex and geography, specifically, between East Asian and Australasia, Europe, and North America. This an observational study encompassing two East Asian states (Japan and Taiwan) and 12 European+ states (Australia, Austria, Denmark, Finland, Germany, Great Britain, Norway, New Zealand, Spain, Sweden, Switzerland, and the USA) over the period 2011–2016. It draws on data compiled from national statistical agencies, vascular registries, and academic publications.

## 2. Methods

### 2.1. Procedures

While national vascular surgical registries have been established in various states, no single organization compiles vascular surgical data on a worldwide basis (for brevity, following WHO nomenclature, “state” is here defined to include “country”). As such, data on EVAR and OSR for the years 2011–2016 were compiled from multiple sources, specifically, government statistical agencies (Australia, Austria, Great Britain, New Zealand, Spain, Switzerland, and Taiwan), vascular registries (Denmark, Japan, Norway, Sweden and the United States), and population-based academic publications (Finland and Germany) (Supplementary Table S1). The years 2011–2016 were chosen to maximize the years of available data across the various states.

### 2.2. Mortality

Data on ruptured AAA mortality (ICD-10 code I71.3), by sex and age for the years 2011–2016, were acquired from the World Health Organization (WHO) database of annual mortality. The WHO database is compiled from reports by member states based on their civil registration systems [12]. In respect to Finland and Taiwan, where the WHO database lacked the appropriate data, the respective government statistical agencies were queried using the same ICD-10 code [13].

The Global Burden of Diseases research group assessed the quality of data on mortality on a 6-point scale ranging from 0–5 stars (GBD 2017, Appendix Figure 5B). Of the states in the present study, seven were rated as 5-star (Australia, Austria, Great Britain, New Zealand, Switzerland, and the United States), and the others were rated as 4-star (Denmark, Finland, Germany, Japan, Norway, Spain, Sweden, and Taiwan).

In order to abstract from differences in the age structure between states, data on population by sex, age group, and year were extracted from the World Development Indicators (WDI) database of the World Bank. As the WDI database did not cover Taiwan, the population data were acquired from the Ministry of the Interior. For each state, sex, age group, and year, ruptured AAA mortality was calculated as the sum of deaths divided by the population. Then, using the proportions in the age groups for the year 2010 [14], age-adjusted ruptured AAA mortality was calculated.

### 2.3. Statistical Analysis

Descriptive analyses of the trends in EVAR prevalence and age-adjusted ruptured AAA mortality, by state and distinguished by sex, were performed.

Manual inspection of the P–P plot suggested that age-adjusted ruptured AAA mortality followed the normal distribution more closely if specified in the natural logarithm. Then, multiple regression analysis was carried out using ordinary least squares for the natural logarithm of age-adjusted ruptured AAA mortality as a function of EVAR, controlling for sex, East Asian states, and indicator variables for each year of the study (Supplementary Materials, Section S3, Equation (S1)). The year indicator variables controlled for global trends in EVAR and ruptured AAA mortality, so abstracted the analysis from the negative correlation between EVAR and mortality.

The model was examined for conformance with the conditions for multiple regression analysis (Supplementary Figure S3). Particularly, the kernel density of the residuals was manually inspected to check that the residuals were normally distributed. To check for heteroscedasticity of the residuals, the residual value plot was manually inspected, and the White’s test was applied. As the null hypothesis of no heteroscedasticity was not rejected (Chi^2^ = 35.13, *p* = 0.5096), standard errors were estimated using the Huber–White robust option, clustered by state. To check for multicollinearity of the explanatory variables, the variance inflation factor was calculated.

The proportionate effect of a 10 percent increase in EVAR on AAA mortality was calculated as the ratio of two variables. The numerator was the exponentiation of 10 multiplied by the estimated coefficient of EVAR, minus 1. The denominator was the average age-adjusted ruptured AAA mortality (prevalence).

Multiple regression analyses were conducted separately for the two sexes to examine differences in the effect of EVAR on mortality by sex, controlling for East Asian states, and including year indicator variables.

Similarly, multiple regression analyses were conducted separately for European+ and East Asian states to examine differences in the effect of EVAR on mortality by state, controlling for sex, and including year indicator variables.

To check the sensitivity of the findings to possible changes in the trends of AAA mortality, the multiple regression analyses were repeated for a sub-sample. Prior research covering 10 of the 14 states in the present study found no changes in the trends of AAA mortality among either females or males in the years 2015–2016 [1]. Accordingly, limiting the analysis to the years 2015–2016 ensured robustness to changes in the trends of AAA mortality. Further, to check the sensitivity of the findings to confound differences in screening across the states, multiple regression analyses were repeated with the inclusion of a variable representing the presence of a screening program in the state.

In all regression analyses, estimates with *p*-values of <0.05 were deemed to be statistically significant and confidence intervals were estimated at 95 percent. All data were analyzed using Stata/MP 17 (Stata, College Station, TX, USA).

## 3. Results

### 3.1. EVAR

Referring to Table 1, in 2011, among females, the proportion of EVAR treatment of ruptured AAAs varied from 0.0 percent in Denmark, New Zealand, and Norway, to 77.8 percent in Taiwan, with the average being 26.1 (s.d. 21.7) percent. By 2016, among females, the proportion of EVAR treatment varied from 8.7 percent in Norway to 93.1 percent in Taiwan, with the average being 43.8 (s.d. 21.6) percent.

**Figure 1:**
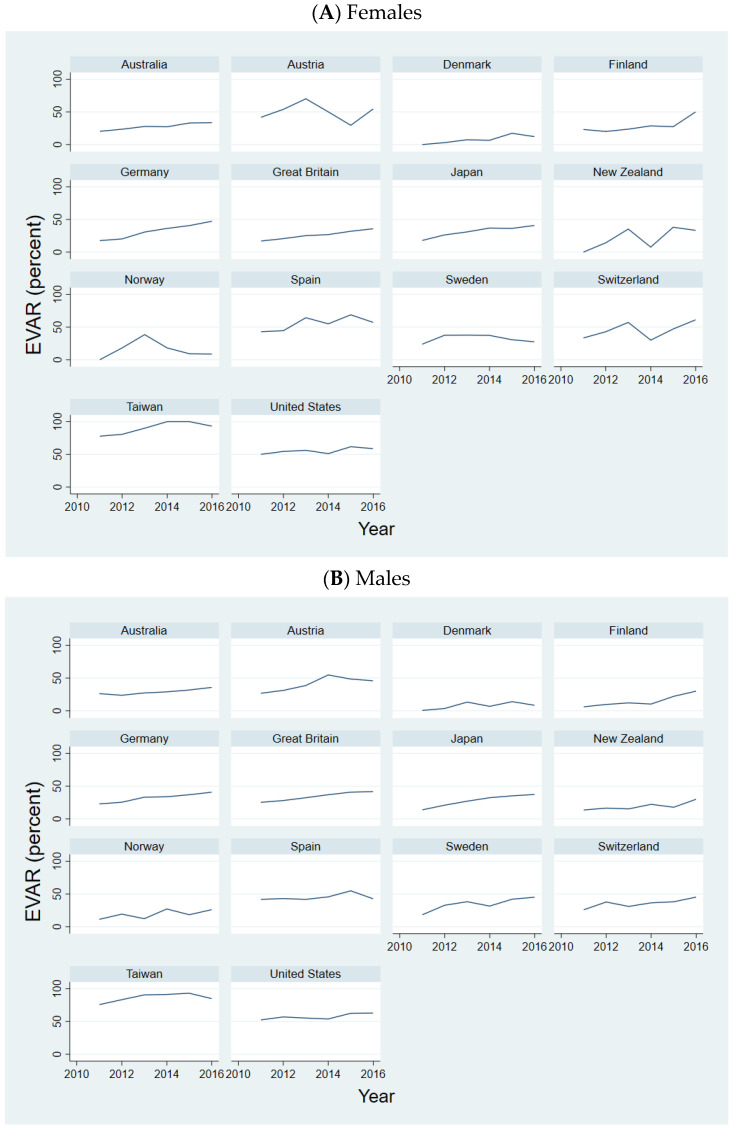
Ruptured AAAs treated with EVAR. Notes: figure depicts the percentage of treatment of ruptured AAAs by EVAR between 2011–2016. (**A**) Female population; (**B**) Male population.

Referring to Figure 1, the overall trend in the treatment of ruptured AAAs among females by EVAR was increasing in all states. Yet, the year-to-year changes varied, and the overall rate of increase varied across the states (Supplementary Figure S1).

Referring to Table 1, in 2011, among males, the proportion of EVAR treatment varied from 0.5 percent in Denmark to 75.8 percent in Taiwan, with the average being 25.7 (s.d. 19.7) percent. By 2016, among males, the proportion of EVAR treatment varied from 8.4 percent in Denmark to 84.9 percent in Taiwan, with the average being 41.2 (s.d. 17.6) percent.

Correspondingly, as Figure 1 illustrates, the overall trend in the treatment of ruptured AAAs among males by EVAR was increasing in all states. Yet, the year-to-year changes fluctuated and the overall rate of increase varied across the states (Supplementary Figure S1).

### 3.2. Ruptured AAA Mortality

Referring to Table 2, in 2011, among females, age-adjusted AAA mortality varied from 2.60 per million in Spain to 47.23 in New Zealand, with the average being 12.62 (s.d. 10.83) per million. By 2016, among females, age-adjusted AAA mortality varied from 2.88 per million in Spain to 30.40 in New Zealand, with the average being 9.50 (s.d. 6.94) per million.

**Figure 2:**
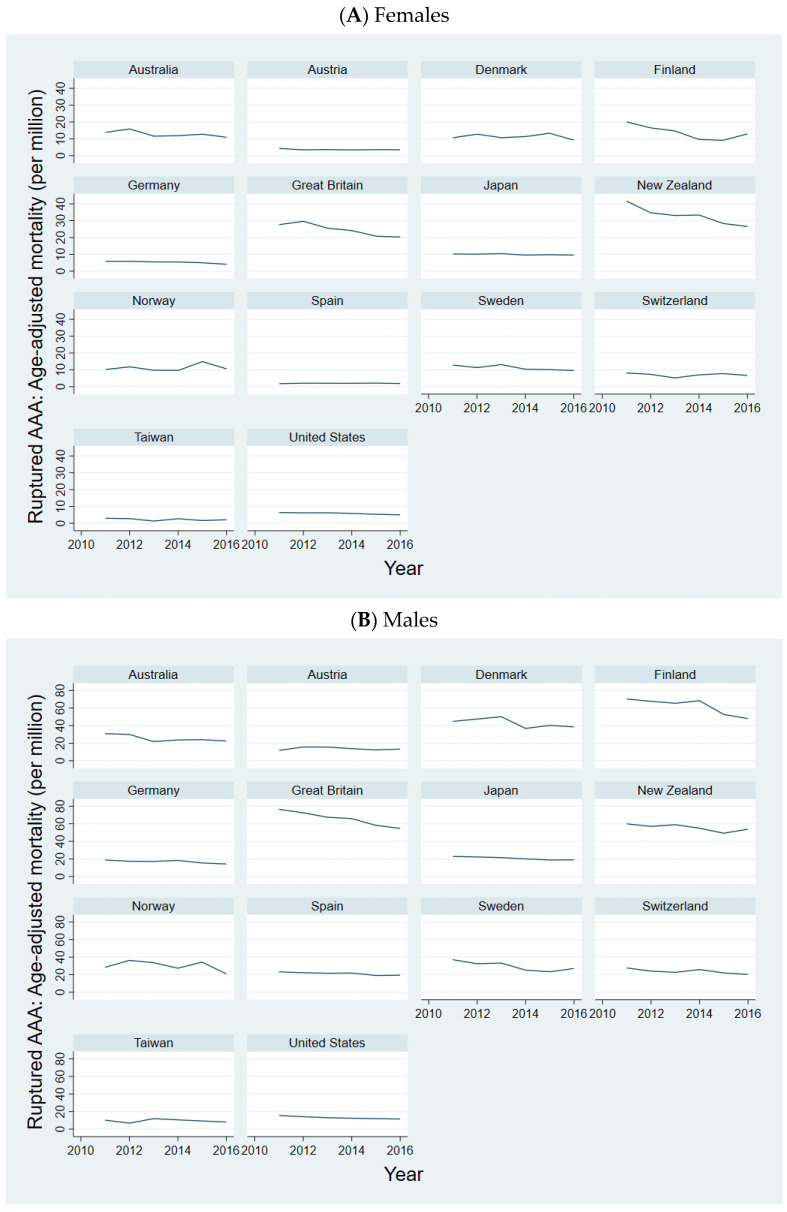
Ruptured AAAs: age-adjusted Mortality. Notes: figure depicts the age-adjusted mortality (per million persons) due to ruptured AAAs between 2011–2016. (**A**): Female population; (**B**): Male population.

Referring to Figure 2, the overall trend in mortality due to ruptured AAAs among females was decreasing in all states except Spain, where mortality was low to begin with. Yet, the year-to-year changes varied, and the overall rate of decrease varied across the states (Supplementary Figure S2).

Referring to Table 2, in 2011, among males, age-adjusted AAA mortality varied from 13.17 per million in Taiwan to 86.87 in Great Britain, with the average being 34.14 (s.d. 21.26) per million. By 2016, among males, age-adjusted AAA mortality varied from 11.79 per million in Taiwan to 64.23 in New Zealand, with the average being 26.54 (s.d. 15.76) per million.

As Figure 2 depicts, the overall trend in mortality due to ruptured AAAs among males was decreasing in all states except Austria. Yet, the year-to-year changes varied, and the overall rate of decrease varied across the states (Supplementary Figure S2).

### 3.3. Ruptured AAA Mortality: Multiple Regression

In the entire sample (Table 3, column (a)), treatment by EVAR and females were associated with lower mortality. Every 10-percentage point increase in treating ruptured AAAs by EVAR was associated with a decrease in age-adjusted mortality of 1.0 (c.i. 0.4 to 1.6) percent of the overall prevalence of 20.90 per million persons.

Among females (Table 3, column (b)), EVAR was associated with lower mortality. Every 10-percentage point increase in treating ruptured AAAs among females by EVAR was associated with a decrease in age-adjusted mortality of 2.2 (c.i. 1.4 to 3.0) percent of the overall prevalence of 11.00 per million females. Among males (Table 3, column (c)), EVAR was associated with significantly lower mortality. Every 10-percentage point increase in treating ruptured AAAs among males by EVAR was associated with a decrease in age-adjusted mortality of 0.6 (c.i. 0.4 to 0.8) percent of the overall prevalence of 30.79 per million males. EVAR was associated with a significantly larger negative effect on ruptured AAA mortality among females than males.

In Europe+ states (Table 3, column (d)), EVAR was associated with lower mortality. Every 10-percentage point increase in the treatment of ruptured AAAs by EVAR was associated with a decrease in age-adjusted mortality by 1.1 (c.i. 0.7 to 1.5) percent of the overall prevalence of 22.61 per million persons. In East Asian states (Table 3, column (e)), EVAR was associated with significantly lower mortality. Every 10-percentage point increase in treatment by EVAR in East Asian states was associated with a decrease in age-adjusted mortality by 1.7 (c.i. 1.7 to 1.7) percent of the overall prevalence of 10.62 per million persons. EVAR was associated with a significantly larger negative effect on AAA mortality in East Asian states than the European+ states.

A possible concern with the findings is changes in the trends of ruptured AAA mortality rates during the study period. Supplementary Table S3 presents multiple regression estimates limited to the years 2015–2016. The results are similar to those reported in Table 3 for the entire study period, 2011–2016. Yet another possible concern with the findings is differences in screening programs across states. Supplementary Table S4 presents multiple regression estimates that control for the presence of a screening program. The results are similar to those reported in Table 3, without such control.

## 4. Discussion

This multi-national study including East Asian as well as European+ states reported consistent decreases in ruptured AAA mortality and increases in the use of EVAR to treat ruptured AAAs. The increases in EVAR were associated with lower mortality, with the reductions being more pronounced among females as compared with males, and in East Asian as compared with European+ states.

The international uptake in the use of EVAR is noteworthy. EVAR was introduced first in Europe and North America, and spread to Asia relatively later. Yet, by the end of the study period, 2016, the use of EVAR among females and males barely crossed 50 percent in northern European countries, whereas it exceeded 85 percent in females and males in Taiwan. As such, EVAR had penetrated widely in Taiwan despite IFU challenges relating to “hostile” aortic necks and smaller iliac artery diameters.

The negative association between EVAR and ruptured AAA mortality is also noteworthy. Early trials and large cohort studies did show that EVAR resulted in lower perioperative and early postoperative mortality for ruptured AAAs [4,5]. EVAR has also extended the option for AAA repair to those who would otherwise have been deemed not to be fit for open surgery. With the increasing availability of EVAR and incorporation into the treatment algorithms of ruptured AAAs, it likely that the pool of patients who are surgical candidates is expanding and as a result, more aneurysms are being repaired in people who might not have otherwise undergone or tolerated OSR [15,16].

Perhaps more surprising is that, despite IFU criteria being less favorable to females and Asians, the negative association between EVAR and AAA mortality was more pronounced among females than males, and more so among East Asian states than European+ states [17]. This difference is possibly due to EVAR being incorporated into clinical practice later in females and East Asia. As the use of EVAR progresses in females and East Asian states, diminishing marginal returns may set in, and the effects on mortality would converge to those among males and European+ states.

Still, implanting EVARs outside IFU remains a topic of debate, as there is conflicting data regarding whether it truly results in statistically increased operative mortality, particularly if adjunctive techniques are used [18,19]. This argument was the basis for the prospective LUCY trial, which did show that when a low-profile stent-graft was used, comparable outcomes could be obtained between males and females. There has yet to be a comparable study looking across ethnic boundaries, which could be a worthwhile endeavor given that a 2019 Japanese study encompassing 51,380 EVARs showed that almost half of their EVARs were performed outside of the IFU [11].

With these data in hand, AAA screening practices may be worth revisiting. A 2019 review of fatal ruptured AAAs in the USA revealed that 43 percent of all fatal ruptures occurred in patients who were not eligible for screening, with women comprising 79 percent of that cohort [20]. Unsurprisingly, there have already been calls to expand screening criteria to include women who have risk factors for AAA development, which the SVS guidelines from 2019 do reflect [21]. Furthermore, several population-based studies in Asian jurisdictions suggest that screening programs may not be indicated given low prevalence [22]; however, these all used the size criteria of 3.0 cm for an AAA. It is known that, compared to Caucasian Americans, Chinese, African, and Hispanic Americans have significantly narrower infrarenal aortic diameters [23]. It is also worth noting that in a study of 5400 elderly patients in China, the mean maximal infrarenal aortic diameter was 1.50 cm, which, if taken as a true population mean, would result in a size definition of 2.25 cm for an AAA using the 50 percent criterion [24]. Thus, using the lower repair threshold of AAA in females as an example [25], further investigation on the appropriate size and subsequent repair criteria in non-white populations would be useful, particularly since our study suggests that EVAR has a larger impact on AAA mortality rates in females and East Asian states.

## 5. Limitations

This study is subject to two limitations. First, as with population-based studies, the data are not individualized. Patient characteristics were limited to aggregates, and key variables such as aneurysm diameter at repair could not be evaluated, which is relevant as it has been previously shown that there is considerable international variation in repair thresholds for AAAs [5]. From a procedural standpoint, it is worth considering that not all EVARs are the same; the brands and models of devices and their availability across the various states would have varied with multiple factors, including surgeon training, timing of commercial approval, and hospital inventory constraints.

Second, the procedural data were acquired from diverse sources, ranging from national registries such as in the case of the United States to insurance claims databases such as in Taiwan. Furthermore, different states updated their EVAR procedural codes at different timepoints (likely related to the timing of commercial availability of the technology in the respective states), which would result in varying degrees of accuracy in capturing the rates of EVAR vis-à-vis OSR. It has also been shown that ICD coding can result in the over-estimation of AAAs [26]. As an extension of this methodology, our mortality data will not discriminate between early postoperative mortality, late postoperative mortality and non-operative mortality.

## 6. Conclusions

The international adoption of EVAR for the treatment of ruptured AAAs coincided with an overall decline in ruptured AAA mortality rates, with the relationship between EVAR and mortality being more pronounced among females compared with males, and more in East Asian states compared with European+ states. Further research should investigate and address such disparities.

## Figures and Tables

**Table 1:** Ruptured AAAs treated with EVAR.

State	Females, 2011	Females, 2016	Males, 2011	Males, 2016
Australia	20.2	33.4	26.2	35.7
Austria	41.7	54.5	26.8	45.8
Denmark	0.0	12.1	0.5	8.4
Finland	23.1	50.0	6.1	30.0
Germany	17.6	47.3	22.9	40.7
Great Britain	17.0	35.8	25.2	41.7
Japan	17.9	40.8	13.8	37.2
New Zealand	0.0	33.3	13.6	30.0
Norway	0.0	8.7	11.3	26.1
Spain	42.9	57.1	41.8	42.6
Sweden	23.8	27.5	18.1	45.0
Switzerland	33.3	61.1	25.8	45.2
Taiwan	77.8	93.1	75.8	84.9
United States	50.0	58.7	52.5	62.9
Mean	26.1	43.8	25.7	41.2
Std deviation	21.7	21.6	19.7	17.6

Notes: Table reports percentage of treatment of ruptured AAAs by EVAR.

**Table 2:** Ruptured AAAs: age-adjusted mortality.

	Females, 2011	Females, 2016	Males, 2011	Males, 2016
Australia	19.37	15.50	40.61	31.08
Austria	5.03	4.61	14.82	16.08
Denmark	16.45	12.87	56.73	45.26
Finland	21.32	13.69	71.91	50.27
Germany	7.71	6.13	24.63	20.40
Great Britain	32.55	23.05	86.87	62.47
Japan	11.75	10.90	26.50	22.11
New Zealand	47.23	30.40	68.35	64.23
Norway	16.62	12.98	47.32	28.93
Spain	2.60	2.88	32.88	28.84
Sweden	13.84	11.11	41.92	29.53
Switzerland	9.58	8.01	38.03	24.14
Taiwan	4.46	3.22	13.17	11.79
United States	10.77	8.55	22.98	17.54
Mean	12.62	9.50	34.14	26.54
Std deviation	10.83	6.94	21.26	15.76

Notes: Table reports age-adjusted mortality (per million persons) due to ruptured AAAs.

**Table 3:** AAA mortality: effect on EVAR by sex and ethnicity.

Variables	(a) Entire Sample		(b) Females		(c) Males		(d) Europe+ States		(e) East Asian States	
	Coefficient (c.i.)	*p*-Value	Coefficient (c.i.)	*p*-Value	Coefficient (c.i.)	*p*-Value	Coefficient (c.i.)	*p*-Value	Coefficient (c.i.)	*p*-Value
EVAR	−0.025 (−0.033, −0.016)	0.001	−0.028 (−0.041, −0.016)	0.001	−0.021 (−0.029, −0.012)	0.001	−0.028 (−0.041, −0.015)	0.001	−0.019 (−0.020, −0.019)	0.002
Female	−1.088 (−1.314, −0.861)	0.001					−1.093 (−1.342, −0.845)	<0.001	−1.023 (−6.569, 4.524)	0.257
East Asian	−0.024 (−0.442, 0.394)	0.905	0.122 (−0.373, 0.618)	0.603	−0.181 (−0.683, −0.322)	0.025				
Year indicator variables	Yes		Yes		Yes		Yes		Yes	
Observations	168		84		84		144		24	
R-squared	0.723		0.543		0.558		0.683		0.912	
States	14		14		14		12		2	
Average mortality (prevalence)	20.90		11.00		30.79		22.61		10.62	
EVAR effect	−0.010		−0.022		−0.006		−0.011		−0.017	
c.i.	(−0.013, −0.007)		(−0.030, −0.014)		(−0.008, −0.004)		(−0.015, −0.007)		(−0.017, −0.017)	

Notes: This table reports multiple regression estimates of AAA mortality by ordinary least squares including indicator variables for years (excluding 2011 as reference), using Stata routine, regress, with robust standard errors clustered by state. The dependent variable is the natural logarithm of age-adjusted AAA mortality (per million persons). Column (a), Global sample: all states, both sexes; Column (b), all states, females; Column (c): all states, males; Column (d): Europe+ states, both sexes; Column (e): East Asian states, both sexes. The EVAR effect is the proportionate change in age-adjusted AAA mortality associated with an increase in EVAR of 10 percentage points.

## Data Availability

The data presented in this study are available on request from the corresponding author. The data are not publicly available due to restrictions that are subject to non-disclosure agreements.

## Supplementary Materials

The following supporting information can be downloaded at: https://www.mdpi.com/article/10.3390/jcm13092464/s1.
