# Supplementary material for: Effect of EVAR on International Ruptured AAA Mortality—Sex and Geographic Disparities
Source: J Clin Med. 2024 Apr 23;13(9):2464. doi: 10.3390/jcm13092464 (PMC11084331; doi:10.3390/jcm13092464)
Supplement: Supplementary file 1 [file jcm-13-02464-s001.zip › jcm-2865223-supplementary.pdf]

Supplement

S1. Data sources

Ruptured AAA deaths: Secondary sources such as the Global Health Data Exchange https://ghdx.healthdata.org/series/global-burden-disease-gbd do not distinguish mortality due to intact vis-à-vis ruptured aneurysms. Accordingly, age-adjusted mortality due to ruptured AAAs by sex (female/male) was calculated from secondary data on deaths and population.

All states except Finland and Taiwan: World Health Organization, www.who.int/healthinfo/statistics/mortality_rawdata/en/. To conform with the procedural data, the data on mortality for Great Britain were limited to England and Wales. Finland: Official Statistics of Finland (OSF): Causes of death, http://www.stat.fi/til/ksyyt/index_en.html. Taiwan: Health Promotion Administration and Statistics Office, Ministry of Health and Welfare.

Population: All states except Taiwan: World Development Indicators, https://data.worldbank.org/. Taiwan: Department of Household Registration Affairs, Ministry of the Interior https://www.moi.gov.tw/files/site_stuff/321/2/year/year.html.

Table S1. Ruptured AAA repair: Open and endovascular

State	Source	Reference
Australia / AUS	Australian Institute of Health and Welfare	Open repair: Procedures data cubes https://www.aihw.gov.au/reports/hospitals/procedures-data-cubes/contents/data-cubes Endovascular repair: requisitioned from Performance Indicators and Quality Unit Health Systems Group, Australian Institute of Health and Welfare
Austria / AUT	Statistik Austria	Requisitioned from Bundesanstalt Statistik Österreich Direktion Bevölkerung Demographie und Gesundheit
Denmark / DNK	Danish Vascular Registry (Landsregisteret Karbase)	Danish Clinical Quality Program – National Clinical Registries; www.rkkp.dk/in-english/

Finland / FIN	Care Register for Health Care, National Institute for Health and Welfare	Laine, Matti T., Sani J. Laukontaus, Reijo Sund, Pekka S. Aho, Ilkka Kantonen, Anders Albäck, and Maarit Venermo, "A population-based study of abdominal aortic aneurysm treatment in Finland 2000 to 2014", Circulation Vol. 136, No. 18 (2017): 1726-1734.
Germany / DEU	Federal Statistical Office (Statistisches Bundesamt)	Olga von Beckerath, Sebastian Schrader, Marcus Katoh, Bernd Luther, Frans Santosa, and Knut Kröger, "Mortality in endovascular and open abdominal aneurysm repair–trends in Germany", Vasa, Vol. 47 No. 1 (2018), 43-48, ESM1.<br>Matthias Trenner, Michael Salvermoser, Albert Busch, Volker Schmid, Hans-Henning Eckstein, Andreas Kühnl, "Mindestmengeneffekte bei der Therapie des abdominalen Aortenaneurysmas", Deutsches Ärzteblatt, Vol. 117, No. 48, 27 November 2020.
Great Britain (England and Wales) / GBR	National Health Service	Hospital Admitted Patient Care Activity: English NHS hospitals https://digital.nhs.uk/data-and-information/publications/statistical/hospital-admitted-patient-care-activity/2018-19 and previous years.
Japan / JPN	National Clinical Database	Japanese Journal of Vascular Surgery, 2011–2017; http://www.jsvs.org/en/
New Zealand / NZL	Australasian Vascular Audit	Khashram, Manar, Ian A. Thomson, Gregory T. Jones, and Justin A. Roake, "Abdominal aortic aneurysm repair in New Zealand: A validation of the Australasian Vascular Audit", ANZ Journal of Surgery, Vol. 87, No. 5 (2017): 394-398.
Norway / NOR	National Vascular Surgery Registry (NORKAR)	NORKAR Årsrapport, 2014-2018 https://stolav.no/fag-og-forskning/medisinske-kvalitetsregistre/norkar/rapporter
Spain / ESP	Institute of Health Information,	Requisitioned from Institute of Health Information (Instituto de Información Sanitaria) https://www.mscbs.gob.es/ Sanidad: Sanidad en datos

	(Instituto de Información Sanitaria)	
Sweden / SWE	Vascular Registry in Sweden (Swedvasc)	Swedvasc Årsrapport, 2006–2017 https://www.ucr.uu.se/swedvasc/arsrapporter
Switzerland / CHE	Office Fédéral de la Statistique	Requisitioned from Office Fédéral de la Statistique OFS Division Santé et affaires sociales www.statistik.admin.ch
Taiwan / TWN	National Health Insurance Research Database	https://nhird.nhri.org.tw/en/index.htm
United States of America / USA	Society for Vascular Surgery: Vascular Quality Initiative	https://www.vqi.org/

S2. Ruptured AAAs -- EVAR and mortality

Figure S1 presents the mean annual changes (in percentage points) in the treatment of ruptured AAAs by EVAR among females and males and the corresponding 95 percent confidence interval for each state.

Figure S1. Treatment of ruptured AAAs by EVAR: annual change

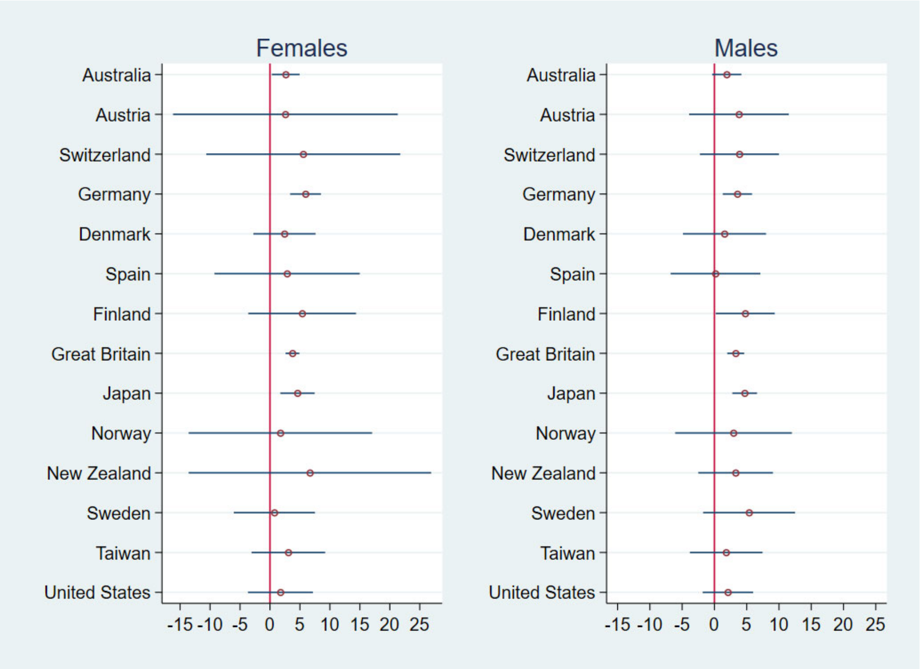

Notes: Figure depicts the mean annual change and 95 percent confidence interval in the percentage of ruptured AAAs treated by EVAR. Left: Female population; right: Male population.

Figure S2 presents the mean annual changes and the corresponding 95 percent CI in the age-adjusted mortality (per million population) due to ruptured AAAs. As the period of study is just six years, from 2011 to 2016, it would be challenging to carry out a meaningful statistical analysis of changes in trends. Previous research showed changes in trends of AAA mortality during the period of study among males for Australia, Denmark, Finland, Germany, and Spain, and among females for Australia, Denmark, Finland, Spain, Sweden, Great Britain (United Kingdom), and the United States (Al-Balah et al., British Journal of Surgery 2020). Accordingly, the annual rates of change should be interpreted with caution.

Figure S2. Age-adjusted mortality due to ruptured AAAs: annual change

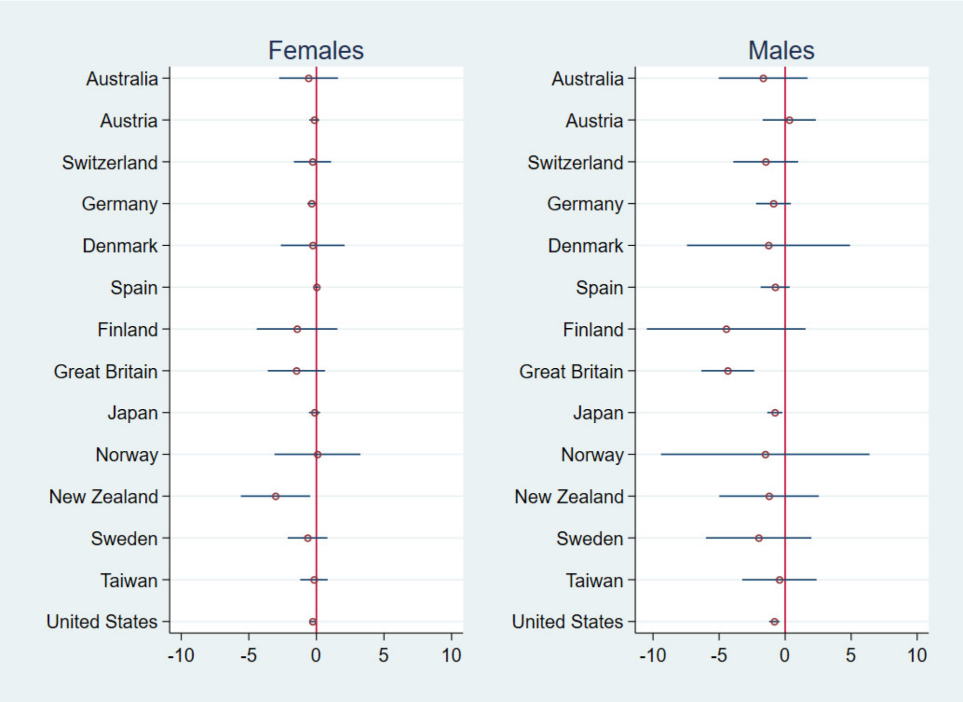

Notes: Figure depicts the mean annual change and 95 percent confidence interval in the age-adjusted mortality (per million persons) due to ruptured AAAs. Left: Female population; right: Male population.

S3. Multiple regression: Supporting information and additional analyses

Formally, the multiple regression equation is, for each state s and year t,

$$ln\,ln\,(MORTALITY_{st}) \;=\; \alpha + \beta_E EVAR_{st} + \beta_F FEMALE_{st} + \beta_A ASIAN_{st} + \beta_t YEAR_t + \varepsilon_{st}. \quad (1)$$

In equation (1), $\ln(MORTALITY_{st})$ represents the natural logarithm of age-adjusted AAA mortality, $EVAR_{st}$ represents the proportion of treatment by EVAR, $FEMALE_{st}$ indicates the mortality of females, $ASIAN_{st}$ indicates mortality in an Asian state, $YEAR_t$ is a vector of indicators of the years 2012–2016, and ε_{st} is statistical error.

In equation (1), the parameters, $\beta_E, \beta_F, \beta_A$, are the coefficients of $EVAR_{st}$, $FEMALE_{st}$, and $ASIAN_{st}$ respectively, and $\beta_t$is a vector of coefficients of the year indicators. The estimated coefficient, β_E, measures the effect of an increase in EVAR by 1 percentage point on the natural logarithm of age-adjusted AAA mortality. Hence, an increase in EVAR by 10 percentage points would be associated with a proportionate change in age-adjusted AAA mortality amounting to

$$\frac{e^{10\beta}E-1}{MORTALITY'} \tag{2}$$

where $MORTALITY$ is the average AAA mortality (prevalence).

To check whether the data meet the conditions for ordinary least squares multiple regression, Figure S3, panel A, presents P-P plots of age-adjusted ruptured AAA mortality in its raw form and specified as a natural logarithm. The distribution of raw mortality did not fit the normal distribution. The distribution of the natural logarithm of mortality closely fit the normal distribution. Accordingly, the multiple regression was estimated with the dependent variable specified as the natural logarithm of mortality. Figure S3, panel B, presents the kernel density of the residuals of the multiple regression. Consistent with the evidence from the P-P plots, the distribution of the residuals closely fits the normal distribution.

Figure S3. Fit with normal distribution

A. Ruptured AAA mortality: P-P Plots

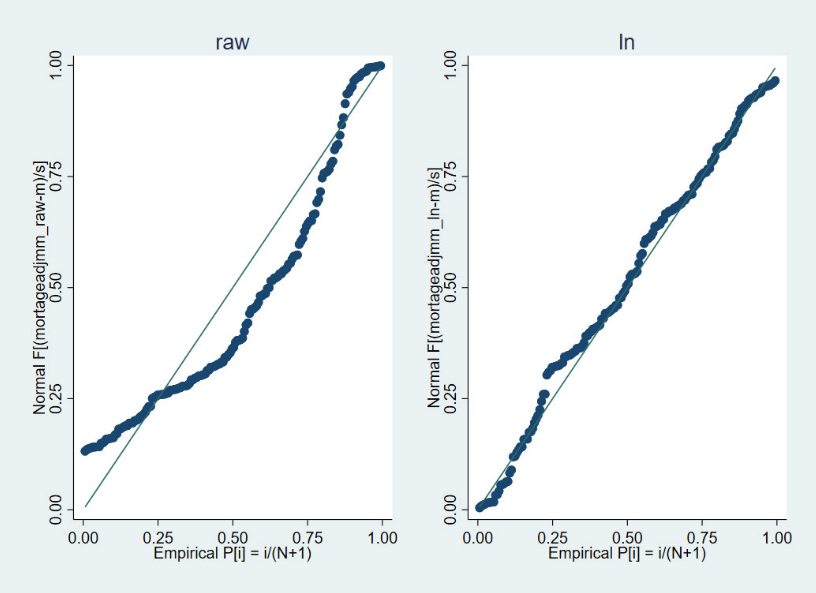

B. Multiple regression residuals: kernel density

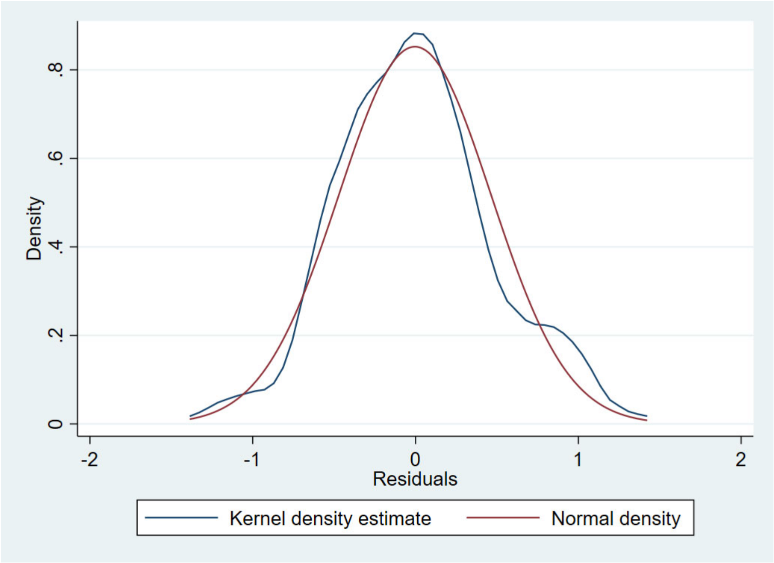

Figure S4 presents an RVF plot of the residuals of the multiple regression against the fitted values of the dependent variable. The residuals appeared to be fairly evenly and randomly distributed above and below the zero line, suggesting the absence of heteroscedasticity. Yet, the White's test could not reject the null hypothesis of no heteroscedasticity (Chi^2 = 35.13, p = 0.5096). Hence, in the multiple regression, the standard errors were estimated using the Huber–White robust option, clustered by state.

Figure S4. Heteroskedasticity

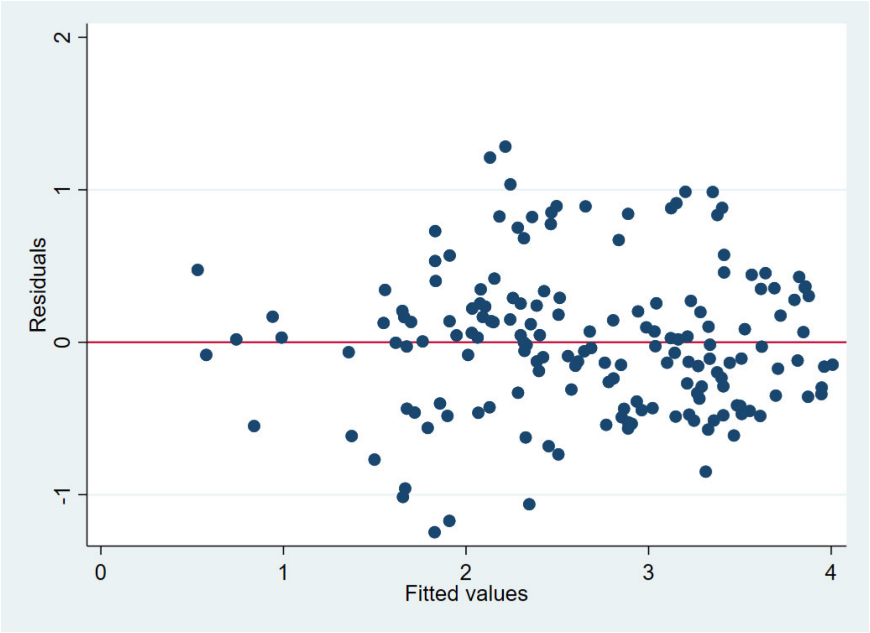

Finally, the mean variance inflation factor is 1.54, which is well within the conventional norm of 5 for absence of multicollinearity.

Table S2 summarizes the data used in the multiple regression analysis.

Table S2. Multiple regression sample: summary statistics

	N	Mean	Std dev	Min	Max
Ruptured AAA mortality (age-adjusted per million)	168	20.90	17.49	1.34	76.48
EVAR (percent)	168	35.8	21.2	0	100
Females	168	0.5	Not meaningful	0	1
East Asian	168	0.143	0.351	0	1

In the main document, Table 1 presents the multiple regression estimates. However, prior research covering 10 of the 14 states in the present study found some changes in the trends of AAA mortality among females and males during the period of the present study (Al-Balah et al. 2020). Table S3 presents estimates limited to the years 2015-2016, during which there were no changes in the trends of AAA mortality among either females or males (Al-Balah 2020: Tables 1 and 2). The results are similar to those reported in Table 1 for the entire study period, 2011-2016.

Another possible concern with the analysis is differences in screening programs across states. During the period of study, Great Britain and Sweden screened men and the USA screened both men and women (Al-Balah et al. 2020; Stather PW, Dattani N, Bown MJ, Earnshaw JJ, Lees TA. International variations in AAA screening. Eur J Vasc Endovasc Surg 2013; 45: 231-234). As for other states in this study, there is no evidence of any screening program. Table S4 presents estimates including an additional explanatory variable to control for the presence of a screening program in the state at the time. The results are similar to those reported in Table 1 without control for screening program.

Table S3. Ruptured AAA mortality: sample limited to 2015–2016

VARIABLES	(a) Entire sample		(b) Females		(c) Males		(d) Europe+ states		(e) East Asian states	
	Coefficient (c.i.)	p-value	Coefficient (c.i.)	p-value	Coefficient (c.i.)	p-value	Coefficient (c.i.)	p-value	Coefficient (c.i.)	p-value
EVAR	-0.024	0.001	-0.026	0.001	-0.021	0.001	-0.035	0.001	-0.022	0.001
	(-0.031, -0.016)		(-0.037, 0.014)		(-0.030, -0.012)		(-0.037, -0.013)		(-0.022, -0.022)	
Female	-1.073	0.001					-1.086	0.001	-0.994	0.297
	(-1.305, -0.841)						(-1.342, -0.831)		(-7.355, 6.366)	
East Asian	-0.032	0.859	-0.081	0.759	-0.153	0.489				
	(-0.410, 0.347)		(-0.476, 0.638)		(-0.617, -0.311)					
Year indicator variables	Yes		Yes		Yes		Yes		Yes	
Observations	56		28		28		48		8	
R-squared	0.709		0.495		0.553		0.644		0.940	
States	14		14		14		12		2	
Average mortality	18.58		9.91		27.24		20.04		9.78	
EVAR effect	-0.011		-0.023		-0.007		-0.011		-0.020	
c.i.	(-0.014, -0.008)		(-0.031, -0.015)		(-0.009, -0.005)		(-0.015, -0.007)		(-0.020, -0.020)	

Notes: This table reports multiple regression estimates of AAA mortality by ordinary least squares including indicator variables for years (excluding 2011 as reference), using Stata routine, regress, with robust standard errors clustered by state. The dependent variable is the natural logarithm of age-adjusted AAA mortality (per million persons). Column (a): Global sample: all states, both sexes; Column (b): all states, females; Column (c): all states, males; Column (d): non-East Asian states, both sexes; Column (e): East Asian states, both sexes. The EVAR effect is the proportionate change in age-adjusted AAA mortality associated with an increase in EVAR by 10 percentage points.

Table S4. Ruptured AAA mortality: control for screening program

VARIABLES	(a) Entire sample		(b) Females		(c) Males		(d) Europe+ states		(e) East Asian states	
	Coefficient (CI)	p-value	Coefficient (CI)	p-value	Coefficient (CI)	p-value	Coefficient (CI)	p-value	Coefficient (CI)	p-value
EVAR	-0.026	0.001	-0.026	0.001	-0.025	0.001	-0.029	0.001	-0.022	0.001
	(-0.035, -0.017)		(-0.040, 0.013)		(-0.037, -0.013)		(-0.043, -0.014)		(-0.022, -0.022)	
Female	-1.021	0.001					-1.017	0.001	-0.994	0.297
	(-1.245, -0.798)						(-1.269, -0.766)		(-7.355, 5.366)	
East Asian	0.087	0.679	-0.103	0.748	0.055	0.852				
	(-0.355, 0.528)		(-0.575, 0.782)		(-0.573, -0.684)					
Screening	0.332	0.233	0.105	0.708	0.409	0.223	0.382	0.185		
	(-0.241, 0.905)		(-0.490, 0.701)		(-0.281, 1.098)		(-0.212, 0.976)			
Year indicator variables	Yes		Yes		Yes		Yes		Yes	
Observations	56		28		56		48		8	
R-squared	0.723		0.578		0.623		0.665		0.940	
States	14		14		14		12		2	
Average mortality	18.58		9.91		27.24		20.04		9.78	
EVAR effect	-0.012		-0.023		-0.008		-0.012		-0.020	
c.i.	(-0.015, -0.009)		(-0.032, -0.014)		(-0.011, -0.005)		(-0.017, -0.007)		(-0.020, -0.020)	

Notes: This table reports multiple regression estimates of AAA mortality by ordinary least squares including indicator variables for years (excluding 2011 as reference), using Stata routine, regress, with robust standard errors clustered by state. The dependent variable is the natural logarithm of age-adjusted AAA mortality (per million persons). Column (a): Global sample: all states, both sexes; Column (b): all states, females; Column (c): all states, males; Column (d): non-East Asian states, both sexes; Column (e): East Asian states, both sexes. The EVAR effect is the proportionate change in age-adjusted AAA mortality associated with an increase in EVAR by 10 percentage points.
